# Dysphagia screening and pneumonia after subarachnoid hemorrhage: Findings from the Chinese stroke center alliance

**DOI:** 10.1111/cns.13822

**Published:** 2022-03-02

**Authors:** Mei‐Ru Wu, Yi‐Tong Chen, Zi‐Xiao Li, Hong‐Qiu Gu, Kai‐Xuan Yang, Yun‐Yun Xiong, Yong‐Jun Wang, Chun‐Juan Wang

**Affiliations:** ^1^ Nursing Department Beijing Tiantan Hospital Capital Medical University Beijing China; ^2^ Vascular Neurology Department of Neurology Beijing Tiantan Hospital Capital Medical University Beijing China; ^3^ China National Clinical Research Center for Neurological Diseases Beijing China; ^4^ Research Unit of Artificial Intelligence in Cerebrovascular Disease Chinese Academy of Medical Sciences Beijing China

**Keywords:** dysphagia, pneumonia, screening, subarachnoid hemorrhage

## Abstract

**Background and Purpose:**

Dysphagia is common and is associated with aspiration pneumonia. However, little is known about the prevalence of and factors influencing dysphagia screening (DS) and pneumonia after subarachnoid hemorrhage (SAH).

**Methods:**

We used data on SAH patients admitted to 1476 hospitals from the China Stroke Center Alliance (CSCA) from August 2015 to July 2019 to analyze the rates of DS and pneumonia. We then conducted univariate and multivariable analyses to examine the relationship between DS and pneumonia.

**Results:**

Among 4877 SAH patients who were eligible for DS and had complete data on pneumonia status, 3527 (72.3%) underwent DS, and 1006 (20.6%) developed pneumonia. Compared with patients without pneumonia, patients with pneumonia were older (mean: 63.4 vs. 57.8 years of age), had lower Glasgow Coma Scale (GCS) scores at admission (mean: 13.5 vs. 14.3), were more likely to have dysphagia (15.2% vs. 3.3%), and were more likely to have undergone aneurysm isolation (19.1% vs. 10.0%). In multivariable analyses, factors independently associated with a higher risk of pneumonia were dysphagia [odds ratio (OR), 3.77; 95% confidence interval (CI), 2.85–4.98], age (OR, 1.50 per 10‐year increase; 95% CI, 1.40–1.60), male sex (OR, 1.23; 95% CI, 1.02–1.49), arrival at the hospital by emergency medical services (OR, 1.36; 95% CI, 1.16–1.58), nimodipine treatment (OR, 1.42; 95% CI, 1.11–1.81), endovascular embolization of aneurysms (OR, 1.23; 95% CI, 1.03–1.47), cerebral ventricular shunt placement (OR, 2.24; 95% CI, 1.41–3.54), and treatment at a higher grade hospital (OR, 1.44; 95% CI, 1.21–1.71).

**Conclusion:**

More than a quarter of patients with SAH did not have documented DS, while one‐fifth developed pneumonia. DS performance was associated with a lower risk of pneumonia. Randomized controlled trials may be needed to determine the effectiveness of DS.

## Graphical abstract

Among 4877 SAH patients who were eligible for DS and had complete data on pneumonia status from the China Stroke Center Alliance, 3527 (72.3%) underwent DS, and 1006 (20.6%) developed pneumonia. Dysphagia was the most significant predictor of pneumonia. In order to reduce the rate of in‐hospital pneumonia among stroke patients, standardized management strategies for dysphagia should be refined.

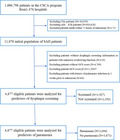

## 1 INTRODUCTION

Subarachnoid hemorrhage (SAH) is a serious and complex disease, with a 30‐day mortality rate ranging from 18% to 40% according to previous literature.^1^, ^2^ Dysphagia is a common focal neurological consequence of SAH that is present in 29% to 77.7% of individuals in the acute phase of recovery.^3^, ^4^, ^5^ Dysphagia is associated with an increased risk of death or dependency, occurrence of pneumonia, poor quality of life, and longer hospital stay.^6^, ^7^ Specifically, dysphagia increases the risk for pneumonia up to 11‐fold and leads to malnutrition, which crucially impedes functional recovery.^8^ Proper assessment of swallowing problems is one of the most important topics in stroke care.^9^ Screening protocols have been developed to identify patients at risk of dysphagia, and earlier screening has been shown to prevent pneumonia.^10^

Despite guidelines advocating dysphagia screening (DS) for all patients with acute stroke,^11^, ^12^, ^13^ little is known about DS practices after SAH and how these practices compare to those for ischemic stroke. In previous studies, seven in 10 patients with SAH were screened, and the rate of failure on DS was approximately 41%. Patients with SAH who fail DS have higher odds of developing pneumonia.^14^ However, there is a lack of data regarding the prevalence of DS and factors influencing the association between DS and pneumonia after SAH.

We used data from the China Stroke Center Alliance (CSCA) to examine (1) the incidence of DS along with factors associated with its application in eligible patients; (2) the incidence in patients with pneumonia and factors associated with pneumonia occurrence; and (3) the relationship between DS and pneumonia in SAH patients.

## 2 METHODS

### 2.1 Study design and target population

The CSCA is a national, hospital‐based, multicenter, voluntary, multifaceted intervention and continuous quality improvement initiative made available to all secondary and tertiary grade hospitals in China. Hospital characteristics, including geographic region, hospital volume (secondary and tertiary grade), and annual stroke volume, were surveyed. The details of this initiative are described elsewhere.^15^ All patients diagnosed with acute ischemic stroke, intracerebral hemorrhage, SAH, or transient ischemic attack (TIA) were included in the CSCA from August 2015 to July 2019. Hospitals intending to join this program contacted the staff of the Chinese Stroke Association voluntarily or were recruited directly by working with the National and/or Provincial Center of Neurological Disease Care Management. The number of hospitals from each province was extracted from the 2016 Statistical Yearbook published by the National Health and Family Planning Commission of the People’s Republic of China.^16^

All target populations of this study should be over 18 years of age and have a primary diagnosis of SAH confirmed by brain CT or MRI. Patients without DS information, unknown swallowing function, GCS scores, GCS scores ≤8, and history of pulmonary infection 2 weeks prior to admission will be excluded from the analysis.

### 2.2 Data collection and management

Professional researchers were systematically trained to review medical records daily at each hospital to identify, consent to, and enroll continuous patients. Data were collected via the web‐based patient data collection and management tool (Medicine Innovation Research Center, Beijing, China),^15^ extracted via chart review, coded, deidentified, and transmitted in a secure manner to maintain patient confidentiality per national privacy standards. All patient identifiers were removed before use in the research, and the use of data for these purposes was closely overseen by the analytic center of the China National Clinical Research Center for Neurological Diseases. The following data were collected for each case: patient demographics, disease and medication history, hospital presentation, initial neurological status, in‐hospital medications and interventions, and in‐hospital outcomes and complications.

### 2.3 Outcomes

DS was defined as the use of a brief noninvasive bedside test by a healthcare professional before any oral intake, including food, liquid, and medication. Considering the consciousness requirement when performing DS, patients with GCS ≤8 points were excluded because of their severe disturbance of consciousness and disability to cooperate with the screening. The 30‐ml water swallowing test was used to screen for dysphagia at all participating hospitals.^17^ Patients were instructed to sit in a relaxed upright posture and drink 30 ml of room temperature water from a cup as able without interruption. According to the time spent drinking the water and the presence or absence of coughing, the results included the following five levels: Level I, drinking water successfully without interruption or coughing; Level II, drinking water with two interruptions and with coughing; Level III, drinking water without interruption but with coughing; Level IV, drinking water over two interruptions and with coughing; and Level V, coughing frequently and cannot drink the water successfully. Swallowing abilities were classified as normal (Level I within 5 s), possible abnormality (Level I over 5 s or Level II), or abnormality (Levels III to V). Possible abnormalities and abnormalities were both considered to indicate dysphagia.^18^ Pneumonia was diagnosed by clinicians according to the Centers for Disease Control and Prevention (CDC) criteria, with support from clinical, biochemical, microbiological, and radiological evidence, such as fever, abnormal chest radiograph, hypoxemia, and the isolation of associated pathogens,^19^ and discharge diagnosis was confirmed on the clinical record.

### 2.4 Statistical analysis

The proportion of patients who underwent DS and had pneumonia was described at the patient level. Continuous variables were presented as the means with standard deviations or medians with interquartile ranges. Categorical variables were presented as counts with percentages. Due to the large sample size, differences that were statistically significant may not be clinically meaningful. We used absolute standardized differences (ASDs) to compare the differences in patients’ baseline characteristics because unlike the *t*‐test, *χ2*‐test, and other statistical tests, ASD is not dependent on sample size; an ASD >10 indicated significant differences between groups.^20^ Multivariable logistic regression models were used to identify patient characteristics that were independently associated with DS and pneumonia. All tests were two‐tailed, and p < 0.05 was considered statistically significant. All statistical analyses were performed using SAS version 9.4 (SAS Institute).

## 3 RESULTS

### 3.1 Case enrollment and target population

**FIGURE 1:**
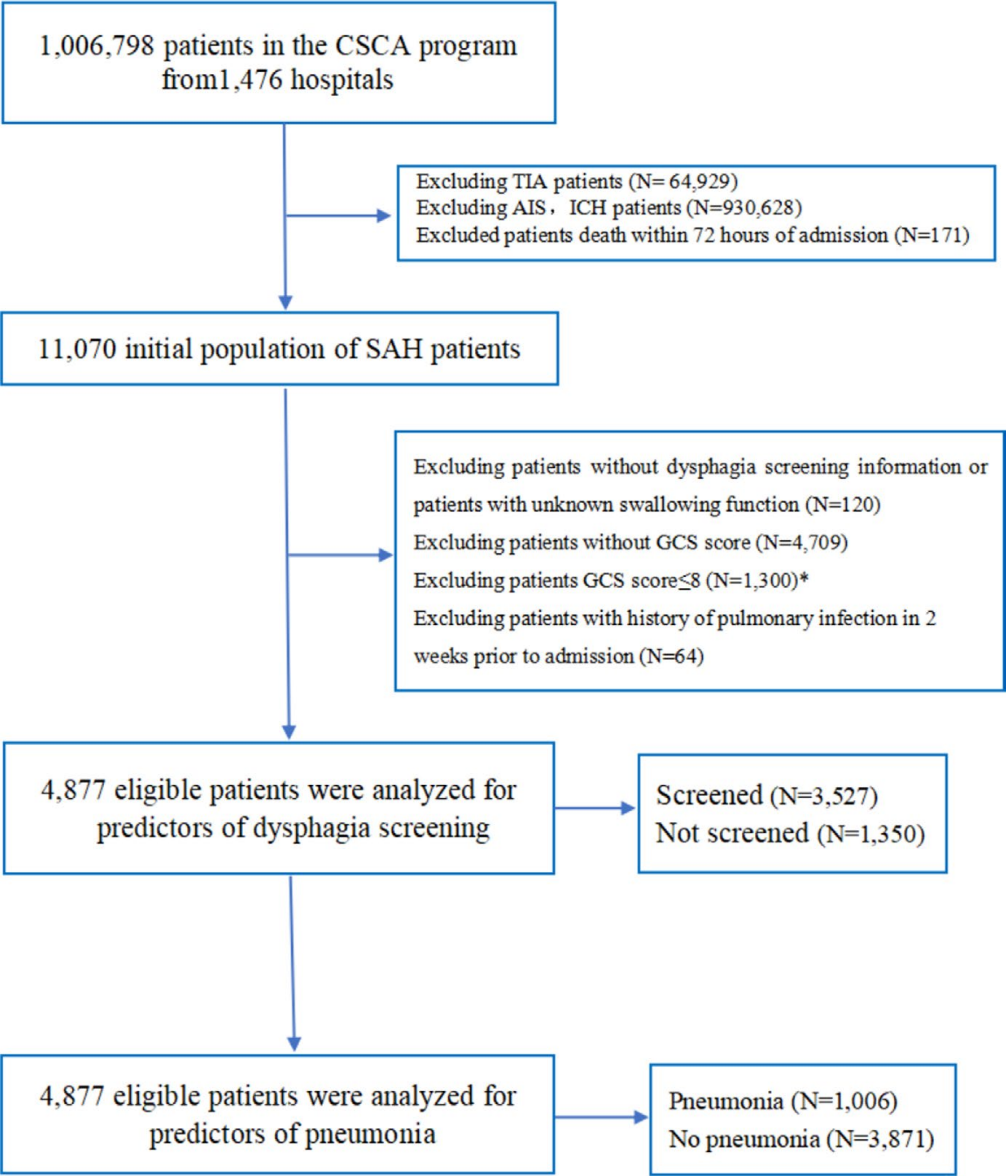
Patient selection. *Patients with SAH with GCS ≤8 points were excluded because they were considered to have severe disturbance of consciousness and could not be screened for swallowing

From August 2015 to July 2019, a total of 1,006,798 patients with acute cerebrovascular events were enrolled in the CSCA, of whom 11,070 (1.1%) were diagnosed with SAH on admission. There was no statistically significant difference in general data between SAH patients with and without GCS scores (Table S1). After excluding patients without DS information, unknown swallowing function, GCS scores, GCS scores ≤8, and history of pulmonary infection 2 weeks prior to admission (*N* = 6193, 55.9%), the final number of patients for this study was 4877 (Figure 1).

### 3.2 Predictors of dysphagia screening after SAH

The study sample included 4877 patients with SAH, among whom 41.5% were male, the mean age was 59.0 ± 12.6 years, and the mean recorded initial GCS score was 14.1 ± 1.6. Of all patients with SAH, 3527 (72.3%) had documented DS. Among patients with mild SAH (GCS score of 14 or 15), the rate of DS was 81.3%. Compared with patients who did not undergo DS, patients who underwent DS (40.7% vs. 55.3%) were less likely to have arrived at the hospital by emergency medical services (EMS) (*p* < 0.0001), and a higher proportion of patients (25.8% vs. 12.1%) were admitted to a hospital in the Western region of China. In addition, patients who underwent DS had higher admission Glasgow Coma Scale (GCS) scores (14.2 ± 1.5 vs. 13.9 ± 1.7) (Table 1).

**TABLE 1:** Baseline characteristics and in‐hospital care of patients hospitalized with subarachnoid hemorrhage who did or did not have documented dysphagia screening

Variables	Total (*N* = 4877 [100%])	Documented screening (*N* = 3527 [72.3%])	No documented screening (*N* = 1350 [27.7%])	*p* value	ASD
Patient characteristics					
Age (y, mean ± SD)	59.0 ± 12.6	59.1 ± 12.6	58.7 ± 12.5	0.2957	3.2
Sex (*n*, %)				0.4052	
Male	2026 (41.5)	1478 (41.9)	548 (40.6)		2.6
Female	2851 (58.5)	2049 (58.1)	802 (59.4)		2.6
Arrival by EMS (*n*, %)	2181 (44.7)	1434 (40.7)	747 (55.3)	<0.0001	29.5
Admission location (*n*, %)				0.0753	
Stroke unit	838 (17.2)	627 (17.8)	211 (15.6)		5.9
Not stroke unit	4039 (82.8)	2900 (82.2)	1139 (84.4)		5.9
In‐hospital measures of health status					
Admission GCS score (Mean ± SD)	14.1 ± 1.6	14.2 ± 1.5	13.9 ± 1.7	<0.0001	18.7
Admission GCS score (*n*, %)				<0.0001	
9–13	1030 (20.9)	666 (18.7)	364 (26.8)		19.4
≥14	3894 (79.1)	2902 (81.3)	992 (73.2)		19.4
Medical history (*n*, %)					
Stroke/TIA	876 (18.0)	608 (17.2)	268 (19.9)	0.0334	7.0
Hypertension	2322 (47.6)	1650 (46.8)	672 (49.8)	0.0609	6.0
Diabetes mellitus	307 (6.3)	213 (6.0)	94 (7.0)	0.2346	4.1
Dyslipidemia	112 (2.3)	79 (2.2)	33 (2.4)	0.6696	1.3
CHD or previous MI	192 (3.9)	138 (3.9)	54 (4.0)	0.8884	0.5
Atrial fibrillation	33 (0.7)	24 (0.7)	9 (0.7)	0.9581	0.0
Carotid stenosis	8 (0.2)	3 (0.1)	5 (0.4)	0.0276	6.0
Peripheral vascular disease	34 (0.7)	29 (0.8)	5 (0.4)	0.0897	5.2
Heart failure	13 (0.3)	9 (0.3)	4 (0.3)	0.8032	0.0
Dementia	4 (0.1)	4 (0.1)		0.2158	
Smoking	1093 (22.4)	794 (22.5)	299 (22.1)	0.7851	1.0
Alcoholism	832 (17.1)	618 (17.5)	214 (15.9)	0.1653	4.3
Hospital characteristics (*n*, %)					
Region				<0.0001	
Eastern	2024 (41.5)	1494 (42.4)	530 (39.3)		6.3
Central	1779 (36.5)	1123 (31.8)	656 (48.6)		34.8
Western	1074 (22.0)	910 (25.8)	164 (12.1)		35.5
Hospital grade				0.0031	
Secondary	1494 (30.6)	1123 (31.8)	371 (27.5)		9.4
Tertiary	3383 (69.4)	2404 (68.2)	979 (72.5)		9.4

In multivariable analyses, factors independently associated with DS included a higher GCS score (OR, 1.11 per point increase in GCS; 95% CI, 1.06–1.15) and treatment at a lower grade hospital (OR, 0.77; 95% CI, 0.67–0.89). Compared to admission to a hospital in the Western region of China, admission to a hospital in the Eastern region (OR, 0.50; 95% CI, 0.41–0.61) or Central region (OR, 0.32; 95% CI, 0.26–0.39) of China was independently associated with a lower likelihood of undergoing DS (Table 2).

**TABLE 2:** Multivariable models of factors associated with documentation of dysphagia screening among SAH patients

Variable	OR (95% CI)	*p* value
Admission location		
Stroke unit versus Not stroke unit	1.06 (0.89, 1.27)	0.5168
Medical history (Yes vs. No)		
Stroke/TIA	0.97 (0.82, 1.14)	0.696
Hypertension	0.92 (0.81, 1.05)	0.2032
Diabetes mellitus	0.94 (0.72, 1.21)	0.6157
Atrial fibrillation	1.17 (0.53, 2.56)	0.698
Smoking	1.02 (0.87, 1.19)	0.8347
Hospital grade		
Tertiary versus Secondary	0.77 (0.67, 0.89)	0.0004
Region		
West (Reference)		
Eastern	0.50 (0.41, 0.61)	<0.0001
Central	0.32 (0.26, 0.39)	<0.0001
GCS score (per 1 point)	1.11 (1.06, 1.15)	<0.0001

### 3.3 Predictors of pneumonia after SAH

Of all patients with SAH who were eligible for DS, 1006 (20.6%) developed pneumonia. Pneumonia was diagnosed in 17.2% (660/3858) of patients with mild SAH (GCS score ≥14). Compared to patients without pneumonia, those with pneumonia were older (63.4 ± 12.2 vs. 57.8 ± 12.4), had lower initial GCS scores (13.5 ± 1.9 vs. 14.3 ± 1.4), and were more likely to have dysphagia (15.2% vs. 3.3%) (Table 3).

**TABLE 3:** Univariate analysis of patient characteristics associated with hospital‐acquired pneumonia among patients eligible for dysphagia screening

Variables	Total (*N* = 4877 [100%])	Pneumonia (*N* = 1006 [20.6%])	No pneumonia (*N* = 3871 [79.4%])	*p* value	ASD
Patient characteristics					
Age (y, mean ± SD)	59.0 ± 12.6	63.4 ± 12.2	57.8 ± 12.4	<0.0001	45.5
Male (*n*, %)	2026 (41.5)	423 (42.0)	1603 (41.4)	0.7148	1.2
Arrival by EMS (*n*, %)	2181 (44.7)	555 (55.2)	1626 (42.0)	<0.0001	26.6
Admission location (*n*, %)				0.3911	
Stroke unit	838 (17.2)	182 (18.1)	656 (16.9)		3.2
Not stroke unit	4039 (82.8)	824 (81.9)	3215 (83.1)		3.2
In‐hospital measures of health status					
Admission GCS score ( Mean ± SD)	14.1 ± 1.6	13.5 ± 1.9	14.3 ± 1.4	<0.0001	47.9
Admission GCS score (*n*, %)				<0.0001	
9–13	1019 (20.9)	346 (34.4)	673 (17.4)		39.6
≥14	3858 (79.1)	660 (65.6)	3198 (82.6)		39.6
Medical history (*n*, %)					
Stroke/TIA	876 (18.0)	207 (20.6)	669 (17.3)	0.0153	8.4
Hypertension	2322 (47.6)	547 (54.4)	1775 (45.9)	<0.0001	17.1
Diabetes mellitus	307 (6.3)	85 (8.4)	222 (5.7)	0.0016	10.6
Dyslipidemia	112 (2.3)	31 (3.1)	81 (2.1)	0.0621	6.3
CHD or previous MI	192 (3.9)	54 (5.4)	138 (3.6)	0.0088	8.7
Atrial fibrillation	33 (0.7)	12 (1.2)	21 (0.5)	0.0250	7.6
Carotid stenosis	8 (0.2)	2 (0.2)	6 (0.2)	0.7597	0.0
Peripheral vascular disease	34 (0.7)	6 (0.6)	28 (0.7)	0.6665	1.2
Heart failure	13 (0.3)	6 (0.6)	7 (0.2)	0.0227	6.3
Dementia	4 (0.1)	2 (0.2)	2 (0.1)	0.1464	2.6
Smoking	1093 (22.4)	246 (24.5)	847 (21.9)	0.0813	6.2
Alcoholism	832 (17.1)	168 (16.7)	664 (17.2)	0.7334	1.3
DS done prior to oral intake (*n*, %)	3527 (72.3)	675 (67.1)	2852 (73.7)	<0.0001	14.5
Swallowing function (*n*, %)				<0.0001	
Normal	3246 (66.6)	522 (51.9)	2724 (70.4)		38.7
Dysphagia	281 (5.8)	153 (15.2)	128 (3.3)		42.0
Treatments					
Nimodipine	4237 (86.9)	902 (89.7)	3335 (86.2)	0.0033	10.8
Endovascular embolization of aneurysms	1539 (31.6)	322 (32.0)	1217 (31.4)	0.7293	1.3
Incarceration of aneurysm	579 (11.9)	192 (19.1)	387 (10.0)	<0.0001	26.0
Cerebral ventricular shunt	91 (1.9)	41 (4.1)	50 (1.3)	<0.0001	17.3
Hospital characteristics (*n*, %)					
Region				<0.0001	
Eastern	2024 (41.5)	344 (34.2)	1680 (43.4)		19.0
Central	1779 (36.5)	436 (43.3)	1343 (34.7)		17.7
Western	1074 (22.0)	226 (22.5)	848 (21.9)		1.4
Hospital grade				<0.0001	
Secondary	1494 (30.6)	242 (24.1)	1252 (32.3)		18.3
Tertiary	3383 (69.4)	764 (75.9)	2619 (67.7)		18.3

After adjusting for age, sex, arrival by EMS, GCS score, hypertension, diabetes mellitus, smoking, DS performed prior to oral intake, swallowing function, hospital grade, and region in patients eligible for DS, we found that variables independently associated with higher odds of pneumonia included dysphagia (OR, 3.77; 95% CI, 2.85–4.98), older age (OR, 1.50; 95% CI, 1.40–1.60), male sex (OR, 1.23; 95% CI, 1.02–1.49), arrival by EMS (OR, 1.36; 95% CI, 1.16–1.58), nimodipine treatment (OR, 1.42; 95% CI, 1.11–1.81), endovascular embolization of aneurysms (OR, 1.23; 95% CI, 1.03–1.47), cerebral ventricular shunt placement (OR, 2.24; 95% CI, 1.41–3.54), and treatment at higher grade hospitals (OR, 1.44; 95% CI, 1.21–1.71). DS performed prior to oral intake (OR, 0.69; 95% CI, 0.58–0.81), GCS score (OR, 0.87 per point increase in GCS; 95% CI, 0.83–0.90), admission to a hospital in the Western region of China and admission to a hospital in the Eastern region of China (OR, 0.71; 95% CI, 0.58–0.87) were among the factors independently associated with a lower risk of pneumonia (Table 4).

**TABLE 4:** Multivariable analysis of patient characteristics associated with hospital‐acquired pneumonia among patients eligible for dysphagia screening

Variable	Adjusted OR (95% CI)^a^	*p* value
DS done prior to oral intake (Yes vs. No)	0.69 (0.58, 0.81)	<0.0001
Swallowing functionDysphagia versus Normal	3.99 (3.03, 5.25)	<0.0001
Age (per 10 years)	1.50 (1.40, 1.60)	<0.0001
Male (vs. Female)	1.23 (1.02, 1.49)	0.0301
Arrival by EMS (Yes vs. No)	1.36 (1.16, 1.58)	<0.0001
Admission location		
Stroke unit versus Not stroke unit	1.06 (0.87, 1.29)	0.5911
Medical history (Yes vs. No)		
Stroke/TIA	0.96 (0.79, 1.16)	0.6829
CHD/previous MI	1.13 (0.79, 1.60)	0.5104
Atrial fibrillation	1.36 (0.62, 2.99)	0.4415
Heart failure	1.93 (0.59, 6.32)	0.2782
Dementia	3.64 (0.36, 37.20)	0.276
Smoking	1.12 (0.90, 1.39)	0.2986
Treatment (Yes vs. No)		
Nimodipine	1.42 (1.11, 1.81)	0.0052
Endovascular embolization of aneurysms	1.23 (1.03, 1.47)	0.0203
Incarceration of aneurysm	2.30 (1.84, 2.87)	<0.0001
Cerebral ventricular shunt	2.24 (1.41, 3.54)	0.0006
Hospital grade		
Tertiary versus Secondary	1.44 (1.21, 1.71)	<0.0001
Region		
Western (Reference)		
Eastern	0.71 (0.58, 0.87)	0.0008
Central	1.01 (0.83, 1.23)	0.924
GCS score (per 1 point)	0.87 (0.83, 0.90)	<0.0001

^a^ Adjusted variables: age, sex, arrival by EMS, GCS score, hypertension, diabetes mellitus, smoking, DS performed prior to oral intake, swallowing function, hospital grade, region.

## 4 DISCUSSION

In this study, we found that 27.7% of patients with SAH were not assessed for DS, with an omission rate of 25.5% among patients with mild SAH. The screening rates in patients with SAH in our study are similar to rates observed in an international multicenter study reporting the frequency of DS to be 69.2% in China, which is lower than that in other countries.^21^ Importantly, patients with mild SAH were most likely to be omitted from screening.

In this study, the DS risk factors in SAH were hospital grade, geographic region, and initial GCS score. These risk factors were similar to risk factors for DS in patients with some degree of stroke. Joundi et al.^10^ reported admission to an ICU or stroke unit and presentation with weakness and speech deficits to be independent predictors of long‐term dysphagia in patients with hemorrhagic stroke. In comparison, age, atrial fibrillation, admission to an academic hospital, geographic region, admission to the ICU or stroke unit, presenting with weakness or speech deficits, and receiving thrombolysis were risk factors for DS in ischemic stroke.^22^, ^23^ Patients with higher GCS scores had less disturbance of consciousness, better understanding and execution of the screener’s instructions, and easier cooperation with DS.

In our study, one‐fifth of the patients developed pneumonia. Pneumonia is a common complication following SAH and has been reported to occur in 20%–49% of patients; a higher incidence has been observed in those undergoing mechanical ventilation.^4^, ^24^ Approximately, 3.5%–21.4% of ischemic stroke patients develop pneumonia, and up to 30% of stroke patients with dysphagia require treatment for pneumonia.^20^, ^25^, ^26^, ^27^ Despite pneumonia occurring more often among patients with more severe stroke, pneumonia occurred even in patients who had high GCS scores at admission (i.e., patients who had strokes of low severity). Our data confirm that these patients are not without a risk of pneumonia since more than one‐sixth of the patients who had GCS scores of 14 or 15 still developed pneumonia.

Treatment for SAH may contribute to the risk of pneumonia. Hypothermia and barbiturates to reduce elevated intracranial pressure may result in immune suppression and decreased leukocyte count, which can predispose patients to pneumonia.^28^ In this study, risk factors for pneumonia included dysphagia, older age, male sex, arrival at the hospital by EMS, nimodipine treatment, endovascular embolization of aneurysms, aneurysm isolation, cerebral ventricular shunt placement, and treatment at a higher grade hospital. In addition, certain risk factors are risk factors for pneumonia in patients with SAH, including the use of mannitol,^29^, ^30^ enteral feeding,^29^, ^30^ and greater height^31^. Our findings on the association between pneumonia severity and older age are consistent with findings in a prior study.^32^ Evidently, patients who arrive via EMS are in more severe distress, and aspiration is common in the early period following SAH. The causes of aspiration pneumonia are manifold, including feeding dependency, oral care dependency, tooth decay, and tube feeding.^33^ Dysphagia increases the risk of aspirating food and oral secretions into the lungs and subsequent pneumonia. Some treatment procedures, such as surgical treatment, increase the patient’s bedtime, which is not conducive to sputum discharge, leading to an increased possibility of pneumonia. Compared to admission to a hospital in the Western region of China, admission to a hospital in the Eastern region of China is more likely to reduce the incidence of pneumonia due to its higher level of medical care, adequate manpower and resources, and rich experience in the prevention and treatment of airway complications. Compared with secondary hospitals, tertiary hospitals have more resources, more thorough professional training for medical staff, and more certified stroke nurses; therefore, tertiary hospitals tend to treat more critically severe SAH patients who have more complications and may have a higher incidence of pneumonia. In this study, admission to the stroke unit was not a factor affecting pneumonia in patients with SAH. To promote stroke units, the national guidelines emphasize that acute stroke patients should be admitted to stroke units, but the development of stroke units is imbalanced. European countries have used stroke units for a long time and have rich experience, and some countries have more than half of stroke patients admitted to stroke units.^34^ China started relatively late, and stroke units still have great room for improvement and can be used in DS process optimization and the management and control of pneumonia.

The observed relationships among dysphagia, aspiration, and pneumonia suggest that conducting DS is an important step in reducing morbidity and mortality. A small before‐and‐after study showed that implementation of DS by nurses reduced the rate of pneumonia as well as the length of stay in the hospital.^35^ In the multivariable analysis, we found that DS performance was associated with a lower risk of pneumonia. Our results differ from those of other studies. One study^20^ reported that DS was associated with an increased risk of pneumonia, while other studies have shown that early screening for dysphagia can effectively identify patients at higher risk of aspiration infection, which is associated with a higher risk of pneumonia, although screening for dysphagia was not associated with a reduction in the incidence of pneumonia or improvements in death or disability in randomized controlled trials (RCTs)^36^, ^37^, ^38^. Notably, in these studies, dysphagia was associated with a higher likelihood of developing pneumonia even in additional sensitivity multivariable analysis that did not include GCS score. As such, conducting DS is a crucial part of preventing pneumonia, and more attention should be given to ensuring that it is included appropriately in clinical pathways for patients being treated for SAH.

### 4.1 Limitations

There were several limitations in our study. First, we gathered data from hospitals covering all regions of China (excluding Hong Kong, Taiwan, and Macao regions), and these hospitals likely used different DS tools. The high rate of missing GCS scores limited our ability to adjust for confounding by SAH severity. The lack of data on antibiotic treatment for the prevention of pneumonia was a failure in the design of the database. The time of initial DS and onset of pneumonia were also not recorded in the database, so we were not able to determine the temporal relationship between initial DS and onset of pneumonia, but the usual time for swallowing screening was on the first day of admission. Some patients may have silent aspiration that is difficult to identify on DS. Although the coding instructions exclude pneumonia present on arrival from the definition of pneumonia, we were able to exclude prevalent cases. Despite these limitations, our study provided a comprehensive characterization of DS and pneumonia in a large SAH cohort.

### 4.2 Conclusion

In summary, more than a quarter of patients with SAH did not have documented DS, and one‐fifth developed pneumonia. DS performance was associated with a lower risk of pneumonia, and the current DS measure may not be optimally designed to elucidate the relationship between screening and pneumonia. In future work, RCTs are needed to determine DS effectiveness and further understand the role of DS in pneumonia prevention.

## CONFLICT OF INTEREST

None.

## AUTHORS’ CONTRIBUTIONS

Conception and design: Chun‐Juan Wang, Yong‐Jun Wang, and Zi‐Xiao Li. Administrative support: Yong‐Jun Wang. Provision of study materials or patients: Zi‐Xiao Li. Collection and assembly of data: Zi‐Xiao Li and Chun‐Juan Wang. Data analysis and interpretation: Hong‐Qiu Gu and Kai‐Xuan Yang. Manuscript writing: All authors. Final approval of manuscript: All authors.

## Supporting information

Table S1
