# Supplementary material for: Dysphagia screening and pneumonia after subarachnoid hemorrhage: Findings from the Chinese stroke center alliance
Source: CNS Neurosci Ther. 2022 Mar 2;28(6):913–21. doi: 10.1111/cns.13822 (PMC9062548; doi:10.1111/cns.13822)

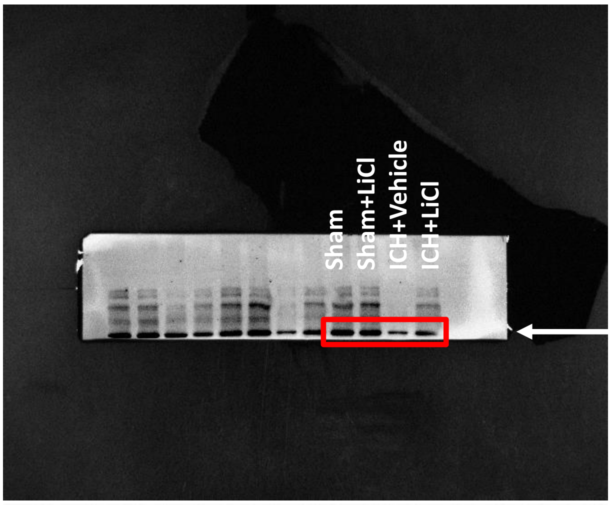

Full unedited blot for

Figure 3A ZO-1

187kDa

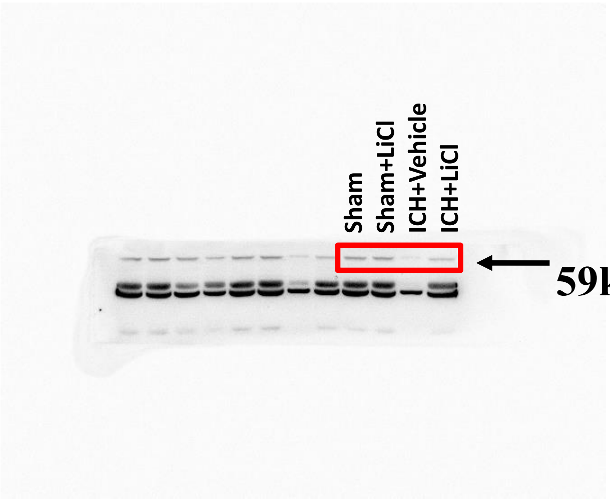

Full unedited blot for

Figure 3A Occludin

59kDa

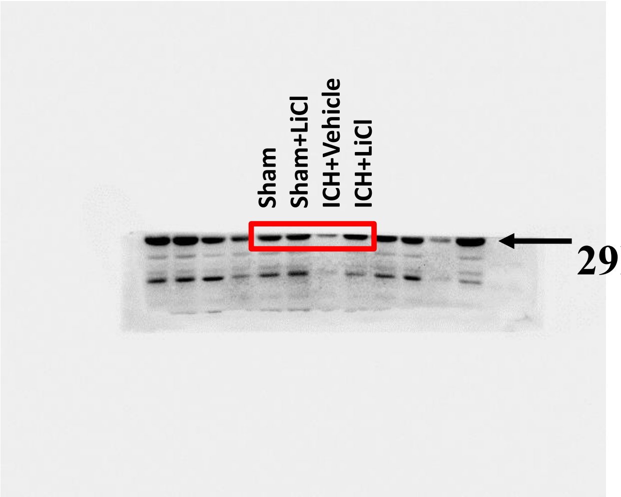

Full unedited blot for

Figure 3A Claudin-5

29kDa

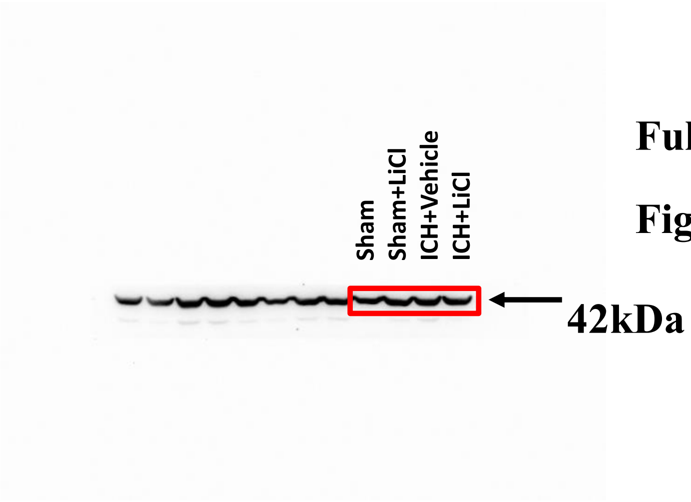

Full unedited blot for

Figure 3A β-actin

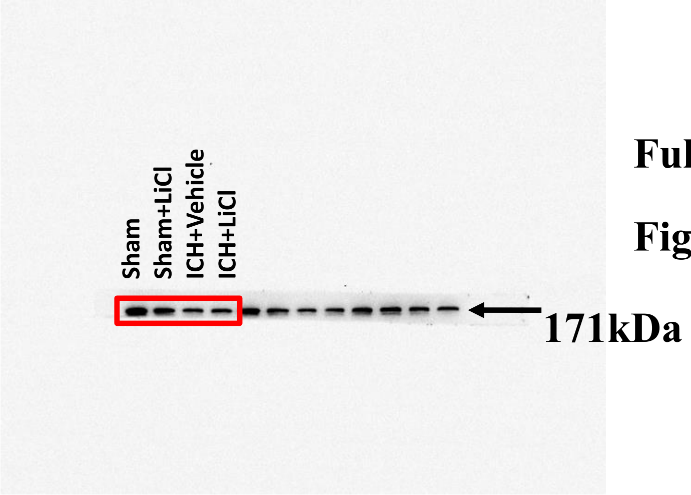

Full unedited blot for

Figure 3A Laminin

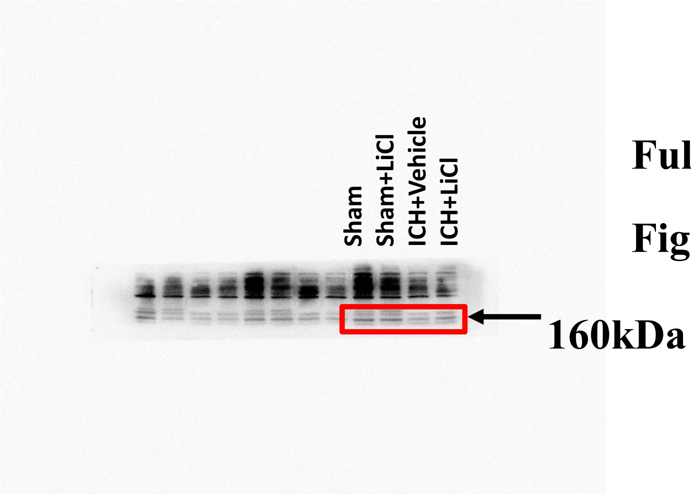

Full unedited blot for

Figure 3A Collagen IV

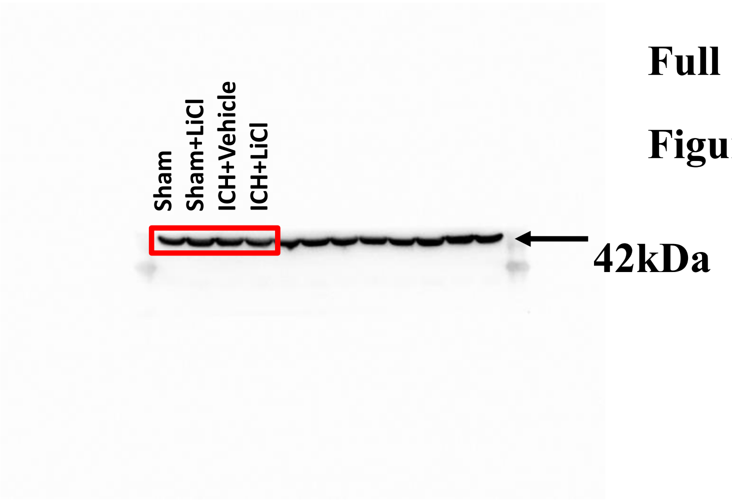

Full unedited blot for

Figure 3A β-actin

Negative staining images with isotype specific IgG is recommended to exclude nonspecific labeling.

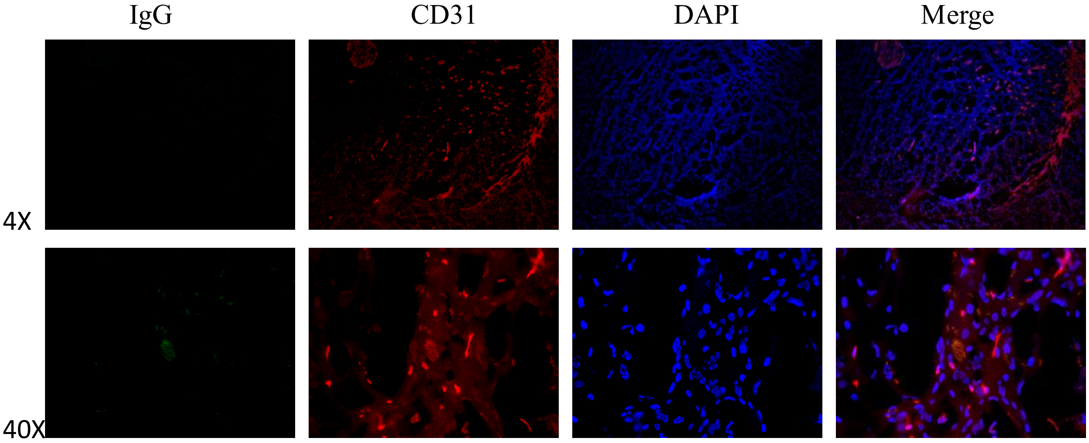

Supplement: Supplementary file 1 — Table S1 [file CNS-28-913-s001.pdf]
